# Complete mitochondrial genome of a Toque Macaque (*Macaca sinica*)

**DOI:** 10.1080/23802359.2018.1437834

**Published:** 2018-02-10

**Authors:** Christian Roos

**Affiliations:** Gene Bank of Primates and Primate Genetics Laboratory, German Primate Center, Leibniz Institute for Primate Research, Goettingen, Germany

**Keywords:** Sanger sequencing, Cercopithecidae, non-human primates

## Abstract

The Toque macaque (*Macaca sinica*) is the only macaque species on Sri Lanka and endemic to the island. The newly generated mitochondrial genome (Genbank accession number MG870385), obtained from a captive individual, has a length of 16,525 bp and exhibits the typical structure of mammalian mitochondrial genomes. Phylogenetically, the Toque macaque is nested within the *Macaca sinica* species group and represents a sister lineage to a *M. assamensis/M. thibetana* clade. The new data help to further illuminate and better understand the complex phylogeny of macaques.

The Toque macaque (*Macaca sinica*) is one of 23 macaque species and occurs only on Sri Lanka (Zinner et al. 2013; Roos et al. 2014). Based on the structure of male external genitalia, the species was lumped, together with *M. assamensis*, *M. thibetana* and *M. radiata*, into the *Macaca sinica* species group (Fooden 1976). The species group further contains *M. munzala* and *M. leucogenys*, two species that were newly described in recent years (Sinha et al. 2005; Li et al. 2015). In a study by Tosi et al. (2003), this species group classification was verified with genetic data, but to date only five partial sequences of the mitochondrial genome of *M. sinica* are available in Genbank, thus calling for additional sequence data of the species.

Consequently, I report here on the sequencing of a mitochondrial genome of a Toque macaque generated from a faecal sample that was collected from a male individual kept at Berlin Zoo, Germany. Total genomic DNA was extracted with the QIAamp DNA Stool Mini Kit following the standard protocol provided by the company (Qiagen, Valencia, CA). The complete mitochondrial genome was PCR amplified, Sanger-sequenced and assembled following methods described in Liedigk et al. (2014, 2015).

The mitochondrial genome has an A + T content of 56.46% and contains 13 protein-coding genes, 22 transfer RNAs, two ribosomal RNAs and the control region in the order typically found in mammals (Anderson et al. 1981).

To trace the phylogenetic position of the Toque macaque, I generated a maximum-likelihood tree using an alignment comprising of 15 macaques and a *Papio hamadryas* used to root the tree. Sequences were aligned with Muscle 3.8.31 (Edgar 2010) in SeaView 4.5.4 (Gouy et al. 2010), and indels and poorly aligned positions were removed with Gblocks 0.91b (Castresana 2000). Tree reconstruction was performed with IQ-TREE 1.5.2 (Nguyen et al. 2015) using the optimal substitution model (TIM2 + I + G) as determined by ModelFinder (Chernomor et al. 2016; Kalyaanamoorthy et al. 2017) and 10,000 ultrafast bootstrap replicates (Minh et al. 2013). In the phylogenetic tree (Figure 1), the Toque macaque is nested within the *M. sinica* species group and forms a strongly supported (100% bootstrap) sister lineage to a clade consisting of *M. assamensis* and *M. thibetana*. These results are in agreement with Tosi et al. (2003) who used a ca. 1.5 kb fragment of the mitochondrial genome, but complete mitochondrial genome data provide stronger node support. The mitochondrial genome of a Toque macaque is an important addition to further illuminate and better understand the complex evolutionary history of macaques.

**Figure 1.**
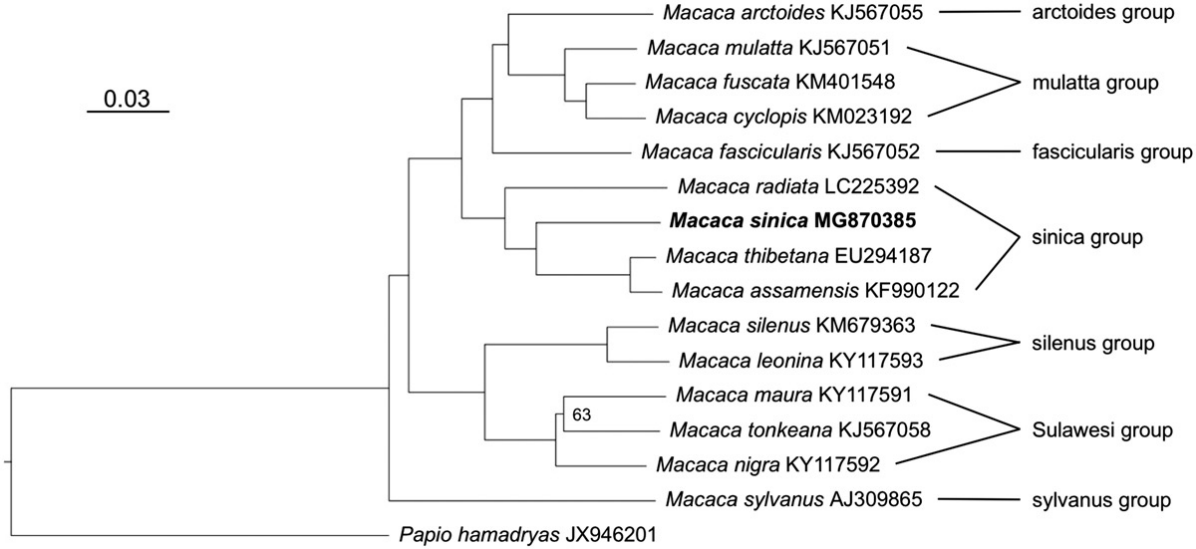
Maximum-likelihood tree displaying phylogenetic relationships among macaques. The Toque macaque, highlighted in bold, is nested within the *M. sinica* species group and is closest related to *M. assamensis* and *M. thibetana*. Node support is generally high with 100% bootstrap support (not shown); only the phylogenetic relationship among the three Sulawesi macaques gained lower bootstrap support (63%). The bar refers to substitutions per site. Genbank accession numbers are given after species names. To the right, macaque species groups are indicated.

## References

[CIT0001] Anderson S, Bankier AT, Barrell BG, de Bruijn MH, Coulson AR, Drouin J, Eperon IC, Nierlich DP, Roe BA, Sanger F, et al 1981 Sequence and organization of the human mitochondrial genome. Nature. 290:457–465. 7219534 10.1038/290457a0

[CIT0002] Castresana J. 2000 Selection of conserved blocks from multiple alignments for their use in phylogenetic analysis. Mol Biol Evol. 17:540–552. 10742046 10.1093/oxfordjournals.molbev.a026334

[CIT0003] Chernomor O, von Haeseler A, Minh BQ. 2016 Terrace aware data structure for phylogenomic inference from supermatrices. Syst Biol. 65:997–1008. 27121966 10.1093/sysbio/syw037 PMC5066062

[CIT0004] Edgar RC. 2010 Quality measures for protein alignment benchmarks. Nucleic Acids Res. 38:2145–2153. 20047958 10.1093/nar/gkp1196 PMC2853116

[CIT0005] Fooden J. 1976 Provisional classifications and key to living species of macaques (primates: Macaca)). Folia Primatol. 25:225–236. 10.1159/000155715 817993

[CIT0006] Gouy M, Guindon S, Gascuel O. 2010 SeaView version 4: a multiplatform graphical user interface for sequence alignment and phylogenetic tree building. Mol Biol Evol. 27:221–224. 19854763 10.1093/molbev/msp259

[CIT0007] Kalyaanamoorthy S, Minh BQ, Wong TKF, von Haeseler A, Jermiin LS. 2017 ModelFinder: fast model selection for accurate phylogenetic estimates. Nat Methods. 14:587–589. 28481363 10.1038/nmeth.4285 PMC5453245

[CIT0008] Li C, Zhao C, Fan P-F. 2015 White-cheeked Macaque (*Macaca leucogenys*): a new macaque species from Medog, Southeastern Tibet. Am J Primatol. 77:753–766. 25809642 10.1002/ajp.22394

[CIT0009] Liedigk R, Kolleck J, Böker KO, Meeijard E, Md-Zain BM, Abdul-Latiff MAB, Ampeng A, Lakim M, Abdul-Patah P, Tosi AJ, et al 2015 Mitogenomic phylogeny of the common long-tailed macaque (*Macaca fascicularis fascicularis*). BMC Genomics. 16:222. 25887664 10.1186/s12864-015-1437-0 PMC4371801

[CIT0010] Liedigk R, Roos C, Brameier M, Zinner D. 2014 Mitogenomics of the Old World monkey tribe Papionini. BMC Evol Biol. 14:176 25209564 10.1186/s12862-014-0176-1 PMC4169223

[CIT0011] Minh BQ, Nguyen MAT, von Haeseler A. 2013 Ultrafast approximation for phylogenetic bootstrap. Mol Biol Evol. 30:1188–1195. 23418397 10.1093/molbev/mst024 PMC3670741

[CIT0012] Nguyen LT, Schmidt HA, von Haeseler A, Minh BQ. 2015 IQ-TREE: a fast and effective stochastic algorithm for estimating maximum-likelihood phylogenies. Mol Biol Evol. 32:268–274. 25371430 10.1093/molbev/msu300 PMC4271533

[CIT0013] Roos C, Boonratana R, Supriantna J, Fellowes JR, Groves CP, Nash SD, Rylands AB, Mittermeier RA. 2014 An updated taxonomy and conservation status review of Asian primates. Asian Primates J. 4:2–38.

[CIT0014] Sinha A, Datta A, Madhusudan MD, Mishra C. 2005 *Macaca munzala*: a new species from western Arunachal Pradesh, northeastern India. Int J Primatol. 26:977–989.

[CIT0015] Tosi AJ, Morales JC, Melnick DJ. 2003 Paternal, maternal, and biparental molecular markers provide unique windows onto the evolutionary history of macaque monkeys. Evolution. 57:1419–1435. 12894949 10.1111/j.0014-3820.2003.tb00349.x

[CIT0016] Zinner D, Fickenscher GH, Roos C. 2013 Family Cercopithecidae (Old World monkeys) In: Mittermeier RA, Rylands AB, Wilson DE, editors. Handbook of the mammals of the world, Volume 3: primates. Barcelona: Lynx Edicions; p. 550–627.

